# Symmetries and asymmetries in the neural encoding of 3D space

**DOI:** 10.1098/rstb.2021.0452

**Published:** 2023-01-30

**Authors:** Kate J. Jeffery

**Affiliations:** Institute of Behavioural Neuroscience, Department of Experimental Psychology, Division of Psychology and Language Sciences, University College London, 26 Bedford Way, London WC1H 0AP, UK

**Keywords:** symmetry, 3D space, spatial cognition, place cells, head direction cells, grid cells

## Abstract

The neural coding of space centres on three foundational cell types: place cells, head direction cells and grid cells. One notable characteristic of these neurons is the symmetry properties of their spatial firing patterns. In symmetric environments, firing patterns are often also symmetric: for example, place cells show translational symmetry in aligned sub-compartments of a multi-compartment environment. A single head direction cell has a mirror-symmetric firing pattern, while a sub-class of head direction cells can show multi-fold rotational symmetries in multi-compartment environments, matching the symmetry of the recently experienced environment. The entorhinal grid cells are notable for the symmetry of their firing patterns in both rotational and translational domains. However, these symmetries are broken in a variety of situations. These symmetry-making and -breaking observations shed light on the underlying computations that generate these firing patterns, and also invite speculation as to whether they may have a functional role. This article outlines these findings and speculates on the consequences of the resultant firing symmetries and asymmetries for spatial coding and cognition.

This article is part of a discussion meeting issue ‘New approaches to 3D vision’.

## 1. Introduction

**Figure 1.**
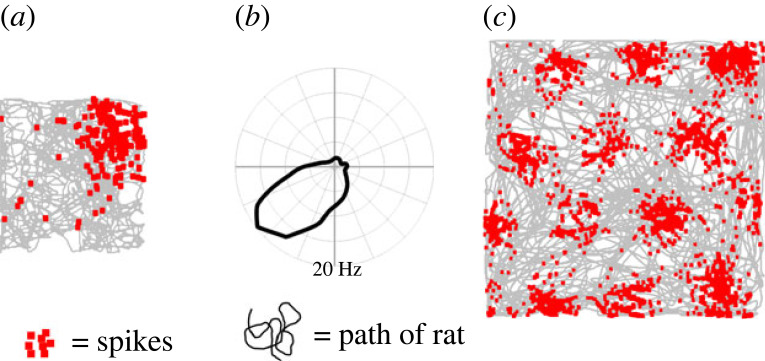
Firing patterns of the three canonical spatial cell types. (*a*) A place cell recorded as a rat explores a square platform typically emits most of its spikes when the animal is in one particular region of the environment. (*b*) A head direction cell fires everywhere (location of firing not shown) but only when the rat faces *n* a particular direction, producing a distinctive firing rate tuning curve. (*c*) A grid cell fires in localized regions of the environment but these are multiple (unlike place cells), are present for a given cell in every environment (also unlike place cells), and are regularly arranged in a symmetric pattern. (Online version in colour.)

The neural code for space centres on three canonical cell types: place cells, head direction cells and grid cells (although many cell types with additional properties or mixtures of properties have since also been found [1]). Place cells (figure 1*a*), originally observed in the hippocampus of rats [2], become active when the animal enters a particular region of the environment, producing spatially localized patches of activity known as place fields or firing fields. Head direction cells fire when the animal faces in a particular direction [3], irrespective of location, producing directionally stable tuning curves. These neurons are found in a number of brain areas, both cortically and subcortically. And finally grid cells, found in entorhinal cortex [4] and pre- and parasubiculum [5], produce circular firing fields that are distributed across the surface of an open two-dimensional environment in a regular close-packed hexagonal pattern.

The discovery of grid cells caught neuroscientists by surprise because neurons with these properties were not predicted by extant models of spatial coding. The amazingly regular, hexagonal, symmetric pattern invites speculation as to the cause and possible functions of symmetries in the spatial firing patterns of the spatial neurons in general, and grid cells in particular. Here, we review the concept of symmetry and the different forms it can take, before examining the ways in which the spatial neurons both form and break symmetries in their spatial patterns. Some speculations on how symmetry and symmetry-breaking could play a role in spatial coding are offered.

## 2. Spatial symmetries

Symmetry refers to the properties of self-similarity possessed by some systems: that is to say, the system (or part of it) is invariant when subjected to some transformation such as translation, rotation, reflection, scaling or a mixture of these. Symmetries can be continuous or discrete: if continuous, the self-similarity holds for an arbitrary movement in a given domain whereas if discrete, it only recurs at intervals.

**Figure 2.**
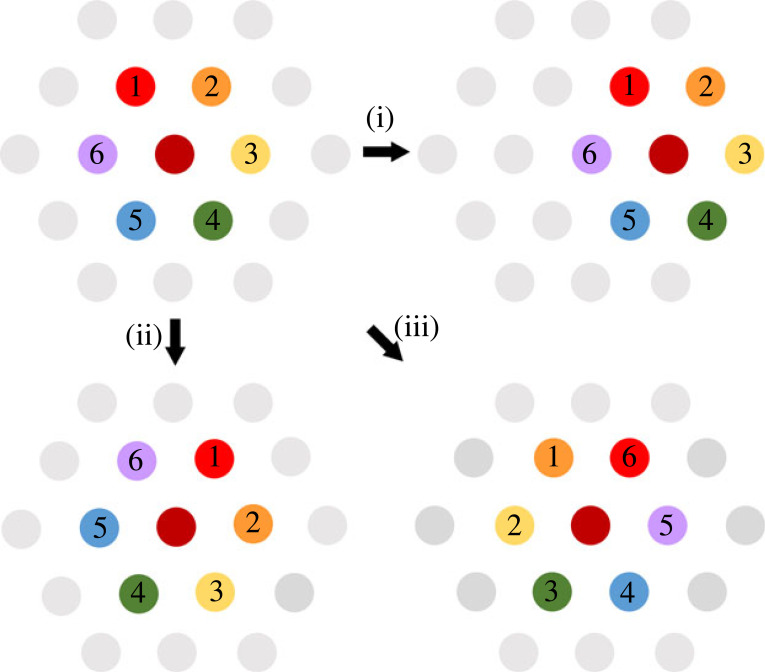
The basic grid cell firing pattern, with fields distinguished for illustration, has three types of symmetry: (i) translational, (ii) rotational and (iii) mirror. (Online version in colour.)

Symmetry plays a fundamental role in physics because it is the invariance of the laws of nature under transformations across space and time that accounts for fundamental physical properties such as the conservation laws of energy, momentum and angular momentum. In the case of the spatial neurons, discovery of the symmetric pattern expressed by grid cells caused a great deal of surprise, since the environment lacks these symmetries and so they must be intrinsic. The symmetries of the grid pattern are translational, rotational and mirror, which is a form of rotational (figure 2). Since there are six 60° directions in the 360 degrees of the horizontal plane, the rotational symmetry order is sixfold (order 6).

The symmetric pattern of grid cell raises questions about what, if anything, this symmetry may be for, and the extent to which there are symmetries in the spatial firing of the other spatial neurons as well. Below, we look at the data on symmetry and symmetry-breaking in the spatial neurons, looking at both rotational and translational symmetries and asymmetries, and then speculate as to how these properties may relate to the encoding of space.

### (a) Place cells

**Figure 3.**
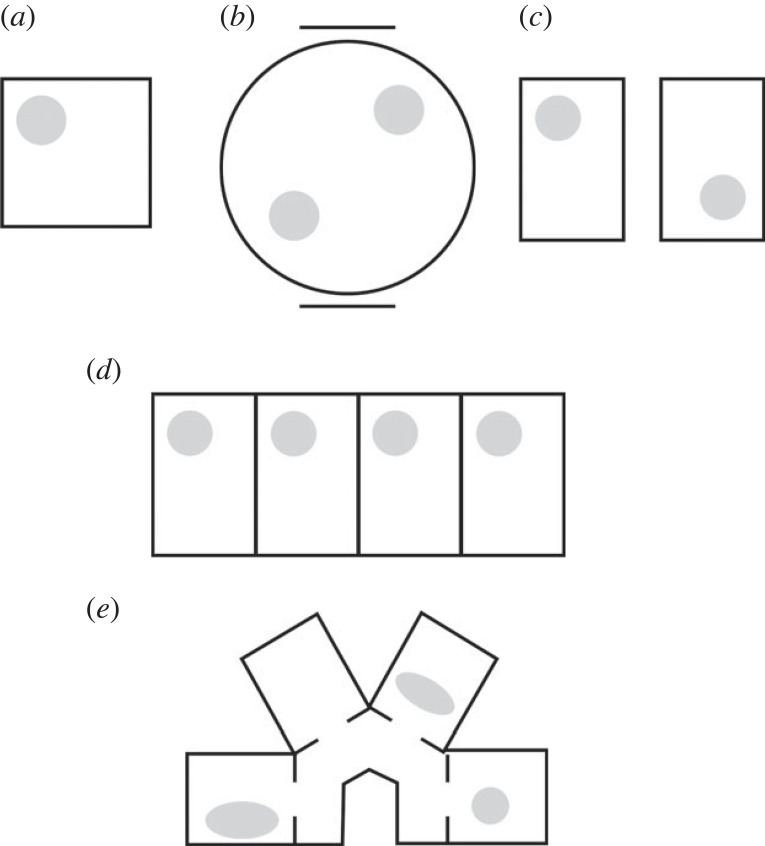
Firing symmetries in place cells. The light grey regions show hypothetical firing fields. (*a*) The basic place cell pattern is asymmetric, inasmuch as the cell fires off-centre in a symmetric environment, pointing to the existence of a symmetry-breaking cue. (*b*) A place cell can produce a field in either of two geometrically equivalent regions of the environment (with geometry constrained by two cue cards at 180° apart, shown by the lines) depending on the entry route of the animal into the environment. (*c*) A place cell will rotate its place field to geometrically equivalent location if the animal's internal sense of direction is manipulated. (*d*) Place cells in connected environments will repeat their fields to follow the environment repetition as long as the compartment orientation is the same. (*e*) If compartment orientation is different, now the fields remap.

The firing of a place cell in an ordinary, simple, bounded arena such as a square box is, in contrast to grid cells, rather *a*symmetric, inasmuch as the cells rarely fire in the exact centre of a symmetric enclosure (figure 3*a*), despite the fact that the boundaries of the enclosure are an important determinant of the location of the firing fields [6]. In a rotationally symmetric enclosure such as a square or circle, the off-centre place field breaks the symmetry such that the rotational order is only onefold – a full 360-degree rotation is needed to map the pattern back onto itself. The sources of this symmetry-breaking still remain to be fully elucidated, but broadly speaking there are two. One is asymmetric visual cues, either local (within the apparatus; for example, a cue card) or distal (from the room). These cues make every direction look unique and thus polarize the environment, and many experiments have shown that rotation of these cues can rotate place fields [7]. The other is the animal's ‘sense of direction,’ which is enough to determine the location of an asymmetric place field even in the absence of visible distal cues. This was shown by two early place cell experiments. In the first [8], place cells were recorded from rats exploring a circular chamber with two identical visual cues placed on the walls 180° apart (figure 3*b*): the two cues together converted the infinite rotational symmetry of a bare-walled environment into a visually two-fold symmetric space. However, place cells did not produce twofold-symmetric firing fields: the symmetry was broken by the route of entry through the curtains taken by the experimenter as they placed the rat in the apparatus, so that the cell produced only a single field in one of the two equivalent locations (with respect to the cue card), with the choice of location determined by the entry point. In the second experiment [9], place fields were recorded in a rectangular chamber surrounded by circular curtains (figure 3*c*). Although the overall environment structure had two-fold rotational symmetry, place fields were asymmetrically located in the usual way: however, the location in the box could be switched from one location to the diametrically opposite one if the rat were removed from the apparatus, placed in a covered container and slowly rotated by 180° so as to reorient its sense of direction. These two experiments show that the rat's internal state—its sense of direction—can break environmental symmetry and allow place cells to fire asymmetrically. As discussed below, this signal arises from processing of self-motion cues, sometimes called idiothetic [10], and arriving via the head direction system.

Place cells are also able to break the symmetry of a two-compartment environment in which each sub-compartment has only a visual onefold symmetry but the symmetry of the overall environment is twofold (figure 3*c*). This was first shown by an experiment in which rats were familiarized with exploring two adjacent square boxes that were polarized by a doorway leading to a connecting corridor [11]. Initially, place fields maintained the same alignment of their fields in the two boxes. The boxes were then rotated by 90°, one clockwise and one counterclockwise, so that the doors now connected the boxes directly. This had the effect of directionally reversing the two environmental polarities such that the doorway for one box was in one direction—say, ‘East’—and in the other box it was in the other direction (West). Place fields initially remained the same in the two boxes (that is, the cells ignored the relatively altered position of the doors) but eventually came to be different. Thus, the cells ‘remapped’ (expressed a new representation). They never, however, rotated to follow the rotated environment layout. More recently, a similar result has been reported for two rectangular boxes joined by a central doorway, which we call here a 2-box. In this apparatus place cells never rotated their fields between the two boxes: some repeated their fields and some remapped ([12]).

The repeating of place fields between one environmental sub-compartment and the next, in situations where the boxes have the same orientation in the room, is a translational symmetry, which echoes the translational symmetry of the environment itself. It suggests that the same environmental information is driving the cells to firing threshold in both environments, while information that might be used to distinguish the compartments, such as self-motion information about distance walked between them, is apparently unavailable (or at least unused). This was shown more extensively in an environment with four parallel adjacent compartments [13] in which place field patterns repeated across all four compartments (figure 3*d*). However, this was only the case if the compartments were aligned in the same direction: if they were rotated, so that the long axis of each box had a different direction, then place fields did not rotate to follow the new alignment: they now remapped [14] (figure 3*e*). These multi-compartment experiments are discussed in more detail by Grieves *et al.* [15].

Overall, these experiments suggest that directional information is the prime symmetry-breaking cue for place cells, such that in similar environments that have the same alignment the cells will produce the same pattern, while in similar environments with different alignment they will remap. It seems that the mismatch between environment layout and global direction is important. It is interesting next, then, to look at firing symmetries in directional neurons.

### (b) Head direction cells

**Figure 4.**
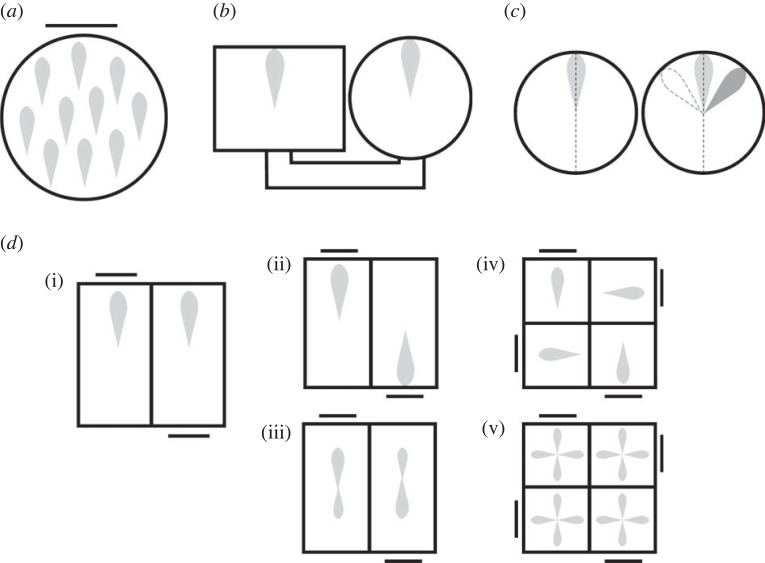
Firing symmetries in head direction cells. The light grey teardrops show hypothetical tuning curves. (*a*) In a chamber with a polarizing cue card, the firing has a continuous translation symmetry in which the firing direction is the same everywhere, and not directed toward the polarizing cue. (*b*) There is also a translational symmetry between two separate chambers, provided the animal can walk between them. (*c*) The tuning curve of a single neuron (left) has a rotational asymmetry but a mirror symmetry around the long axis of the tuning curve (dotted line). When cells with non-overlying tuning curves are considered together (right) then the mirror symmetry breaks down because left and right are not the same. (*d*) Firing patterns in multi-compartment spaces with rotationally symmetric global organization. (i) Classic head direction cells show translational symmetry in the rotationally symmetric 2-box, suggesting an insensitivity to the polarizing visual cues (cue card and central doorway). (ii) A sub-population of directional neurons in dysgranular retrosplenial cortex show singular tuning curves in each sub-compartment, but these follow the environment layout, resulting in a twofold rotational symmetry overall. (iii) A second subset of neurons in this region acquires and retains a twofold rotational symmetry even in each sub-compartment. (iv) The same brain region expresses a similar pattern in a fourfold-symmetric four-compartment space, and (v) some neurons express this fourfold symmetry in each sub-compartment.

The activity of head direction cells on an ordinary flat surface has a continuous translational symmetry, in that the firing direction of a given neuron tends to be the same regardless of the animal's position (figure 4*a*), provided the animal moves of its own volition around the space. This was first shown by observing in single-compartment environments that the firing directions tend to be parallel throughout the chamber and not directed toward the visual cue card that broke the symmetry [3]. This observation was then extended to two-compartment environments in which rats could walk under their own volition from one compartment to another [16] (figure 4*b*), where it was found that firing directions would be aligned provided the second environment was novel when the rat did so (see also [17]). Firing directions of classic head direction cells also remain aligned across two connected compartments even if the visual scenes are rotated with respect to each other [18,19] (but see below for non-classic directional cells). Thus, it seems that self-motion cues can be used to coordinate the signals across the two spaces: something that may be adaptive for navigation. A learning process then occurs that associates the environmental cues to that signal so that those cues alone can reinstate the signal if the animal is disoriented [10].

By contrast with this translational symmetry, the head direction signal has a rotational *a*symmetry: that is to say, it shows only a onefold rotational symmetry, in which each tuning curve, when plotted radially in a polar plot, has to be rotated a full 360° to map to itself. However the firing pattern for a single cell has a mirror symmetry, because it maps to itself if it is reflected (or, equivalently, rotated orthogonal to the plane) around the midline of the tuning curve. However, if more than one cell is considered together, then if their tuning curves are not overlying there is now a mirror asymmetry (figure 4*c*) because a cell that fires to the right of another in the original condition fires to the left in the reflected condition.

These asymmetries are present even when there is visual symmetry in the environment [20]. As with place cells, the symmetry-breaking cues come in two forms: visual landmarks, rotation of which can rotate the firing directions [21], and self-motion cues, which in this domain comprise signals about angular head velocity (arriving from the brainstem) as well as optic flow cues [22,23]. To remain asymmetric when there is visual symmetry, or in the dark with no vision, the system needs to distinguish left-hand from right-hand head turns: this may be accomplished by a population of asymmetric angular velocity-sensitive neurons in the dorsal tegmental nucleus of the brainstem [22], which fire at an increasing rate with increasing velocity in one direction, and at a decreasing rate in the other. Ultimately, the asymmetries in this system are able to arise because of the bilateral mirror symmetry of vertebrates: this means that left turns generate different forces than right turns relative to the anatomy of the vestibular system (particularly the semicircular canals).

An interesting problem for the system arises if the animal inverts—for example, when climbing on the under-surface of a cage top, as mice often do. Now, the global directional consequences of left and right head-turns are reversed—a rightward turn takes the head from facing (say) North to West, instead of to East as in the upright condition. It seems that in rodents, nature may not have found a simple way to cope with this reversal, as inversion results in degradation of the head direction signal [24]. In bats, the head direction cells reverse their global direction so that a cell firing to the North in the upright position fires to the South when inverted [25]—thus, the whole network is essentially flipped upside down, retaining its internal coherence but reversing its relation to the world at large. The system in bats thus has an additional continuous rotational symmetry around the medio-lateral axis of the animal.

In a rotationally symmetric two-compartment environment like the 2-box, classic head direction cells share, with place cells, resistance to producing a rotational symmetry: as mentioned earlier, they don't rotate their firing to follow the rotated environmental cues [19] (figure 4*d*(i)). However, because the overall environment has rotational symmetry, in the absence of symmetry-breaking environmental or self-motion cues the asymmetric firing of the cells would nevertheless randomly fluctuate from one direction to the other between one recording trial and the next since the system has no way to tell which direction is which—they both look the same. For global consistency, some other asymmetric environmental cue needs to break the symmetry. Slightly surprisingly, rotationally asymmetric olfactory cues can serve this function. In an experiment in which one sub-compartment smelled differently from the other (one of lemon and one of vanilla), head direction cells maintained a globally stable firing direction [19], meaning that the relative locations of the lemon and vanilla compartments were able to break the visual symmetry. How this might be done remains unknown, but the finding suggests some type of olfactory-visual integration that causes the polarizing visual cues in a given compartment (the door and the cue card) to become associated with the odour cues, so as to have opposing directional meaning to the system. One possible route for this binding is via the hippocampus, which projects to the head direction system via subicular projections to the granular retrosplenial cortex [26,27].

The firing asymmetries described above pertain to classic head direction cells, found throughout the brain in numerous cortical and subcortical regions [28]. However, in dysgranular retrosplenial cortex there exists a newly discovered second subpopulation of directionally tuned neurons that do actually show a rotational symmetry in the 2-box (figure 4*d*(ii) and (iii))—and also, as recently shown, in a four-compartment version known as the 4-box [29] (figure 4*d*(iv) and (v)). This firing symmetry takes two forms. In the first form, the symmetry applies only to the recording session as a whole, in which animals have explored all the rotated sub-compartments and all the data are considered together. If the firing within single sub-compartments is examined in isolation then the firing has the usual asymmetric pattern of the classic cells (figure 4*d*(ii) and (iv)). The second sub-population is stranger. These cells show rotational symmetries even within single sub-compartments, but only if the animal had previously explored the global space (figure 3*d*(iii) and (v)). Thus, in animals exposed to the 2-box the firing is twofold-symmetric, while if they had explored the 4-box then it is fourfold-symmetric (figure 3*d*(iv) and (v)). Thus, for these cells the global symmetry of the environment has become imprinted onto the local firing pattern. The mechanism for this may involve multiple associations with the classic head direction cells [30], which have different relationships to the environment layout in the different sub-compartments.

These multi-directional cells in retrosplenial cortex form part of a growing collection of landmark-sensitive directionally tuned neurons which have also been reported in parietal cortex [31], medial entorhinal cortex and parasubiculum [32] and postrhinal cortex [33]. These are thought to mediate the learning of associations between environmental layout and global direction [34]. Relatedly, there have been several reports of neurons that show symmetric firing patterns in symmetric environments due to the sensitivity of the neurons to the relative location of boundaries. These so-called egocentric boundary cells become active when the movements of the animal take it to a region of the environment in which there is a boundary at a given distance and direction from the animal [35–37]. The function of these neurons is yet to be determined, but they may be part of a system that acts to transform egocentrically related spatial information to an allocentric spatial map. If so, the symmetric firing in a symmetric environment might be information that a symmetry-detecting system could read out (see Discussion).

### (c) Grid cells and symmetry

The striking symmetry of grid cell firing patterns electrified the spatial coding field when it was first reported in 2005 [4] because it was so unexpected. As mentioned earlier, the grid pattern expressed by a single cell has mirror, translational and sixth-order rotational symmetry, irrespective of the symmetries in the environment (with some qualifications, to be discussed below). The translational symmetry of a canonical grid is discrete: the pattern only maps onto itself after being displaced by a given distance. That distance is different in different directions, but the distribution of these distances has rotational symmetry of order six, due to the rotational symmetry of the pattern itself. If one considers the population of grid cells, rather than single cells, then the distances over which the pattern must be translated in order to map to itself become much greater, because the inter-field spacing of grid cells varies from dorsal to ventral entorhinal cortex [4,38]. Over the typical distances ranged by a normal animal, the ensemble pattern becomes, practically speaking, asymmetric: something that may enable spatial localization.

In the years after the discovery of grid cells, several models were put forward to try and explain how this symmetric firing pattern arises, which can broadly be divided into oscillatory-interference and attractor-based models [39]. In oscillatory interference models [40] the periodicity of the pattern is proposed to arise from the periodicity of rhythmic neuronal firing, converted from the temporal to the spatial domain by a velocity (space/time) signal. In attractor models [41] the periodicity arises because of competition between local excitation and longer range inhibition, with the balance shifting back and forth from one to the other as the animal translates through space.

Carpenter *et al*. found that grid cells also showed a higher level translational symmetry in a novel two-compartment environment, similar to the findings from place cells discussed earlier: that is, the pattern in the two connected environments was the same [42], even though this broke the global symmetry. This suggests that the grid is influenced by the environment boundaries. However, over time the pattern was found to adjust to become more continuous across the global space, suggesting that the conflict between the symmetry conferred by the environment and the symmetry conferred by the local self-motion cues was driving slow plasticity, such that the system could now distinguish the two otherwise identical compartments based on their relative location.

### (d) Symmetry-breaking in grid cells

**Figure 5.**
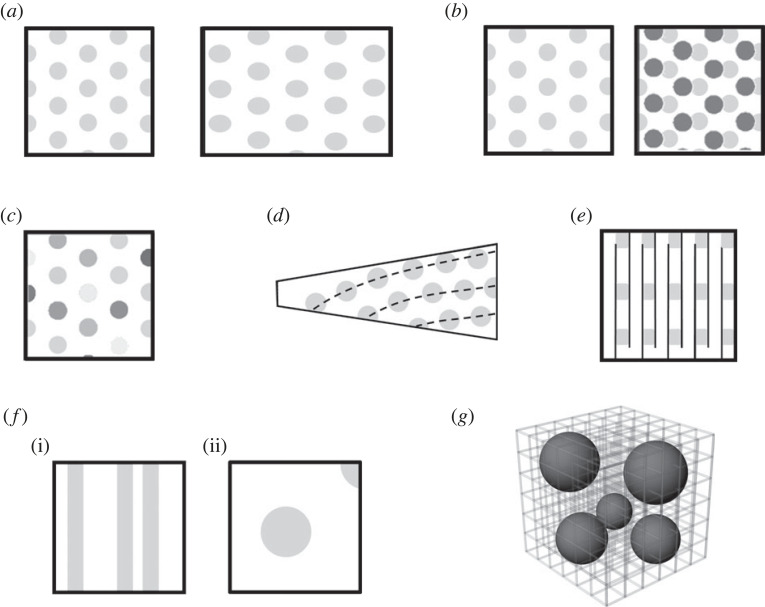
Symmetry-breaking in grid cells. (*a*) Grids can stretch, reducing their symmetry to a single translational symmetry along one dimension, if the environment is stretched in that dimension. (*b*) Grids translate but do not rotate if the environment context is changed but all spatial cues remain the same. (*c*) If firing rate within each sub-field of a grid is taken into account, the symmetry disappears because firing rates are heterogeneous (as shown by the varied shading). (*d*) Grids are distorted even in a novel environment if that environment has an asymmetry. (*e*) The symmetry of the grid becomes translational in one dimension if the environment is broken up into repeating sub-compartments. (*f*) On vertical walls the symmetry of grids is altered. (i) If the rat climbs on pegs the firing fields form vertical stripes, giving the grid continuous translational symmetry along the vertical dimension. (ii) if the animal can climb on the wall with its body parallel to the surface, then the grid fields become circular again and the continuous translational symmetry disappears. (*g*) In a volumetric space the grids lose their symmetries and become irregular.

Despite the striking symmetry of canonical grids, there are now many experiments reporting the breaking of grid symmetry, in response to manipulations of the environment: these findings reveal the interplay between environmental cues and internally generated ones. An example is the stretching of a grid cell's grid if a familiar environment is stretched slightly [43] (figure 5*a*), indicating a rapidly learned association between the environment boundaries and the other factors (the self-motion cues) that position the fields: this enables the breaking of environment symmetry to also break the symmetry of the grid, removing some of the symmetries and leaving just a translational and mirror symmetry along the direction of the stretching. Another example of environmental influence is how grids translate if the environment keeps its shape but changes its non-spatial sensory qualities (colour and odour; figure 5*b*), indicating that the association between boundaries and the grid fields is modulated (or ‘gated’ [44]) by these non-spatial inputs [45]. Interestingly, the grids do not rotate in this situation. This dissociation between translation and rotation might be explained by the dissociable routes in to the system of directional information (via the head direction system) versus linear motion information (the source of which is still not identified). These two information sources, which we assume to be responsible for the rotational and translational symmetry respectively, can be placed in conflict if the local environment is rotated with respect to the global, room environment: in this situation grid cells rotate their grids somewhat [46], and it seems head direction cells may also do so as well, although very subtly [18], indicating that both global and local cues can contribute to directional calculations.

The above findings can be explained by the acquisition of learned information about environment features, but several experiments also show that grid symmetry can be broken even in naive animals, in certain situations. One such asymmetry occurs when one looks closely at the firing rates of the cells in different fields of the grid: these are heterogeneous [47], and so when firing rates are taken into account the pattern no longer has a symmetry. Another asymmetry occurs in the structure of the grid, which distorts when the environment is asymmetric (for example trapezoidal; figure 5*d*) even if the environment is novel, pointing to a pre-configured influence from the walls [48,49].

Another type of symmetry-breaking occurs when movement patterns are disrupted by alterations to the environment. The first report of this was the finding that in a ‘hairpin maze’—a linear track that bends back and forth, allowing the animal to evenly cover a two-dimensional area with a constrained trajectory—the grid fragmented (figure 5*e*), losing its rotational symmetry [50]. It retained a translational symmetry but the direction of translational invariance was now not intrinsic but governed by the environment structure. In other words, the pattern repeated across the repeating segments of the maze, similarly to how place fields and grids did so in the multi-compartment environments described earlier. The grid cells retained the periodicity of their firing in the direction in which the animal was running continuously (along the length of each track) but lost it in the direction in which the running was interrupted (perpendicular to this—which was only run in short, interrupted segments).

A similar observation was made for animals climbing vertically, either on a pegboard (a wall studded with jutting footholds) or on a helical track that enabled animals to climb [51]. In both cases, the grids retained periodicity in the plane of the animal's locomotion (aligned to the horizontal) but lost it in the direction perpendicular to this. Thus, the usual discrete translation symmetry of the grids was converted to a continuous one (the grid mapping onto itself at every incremental step) and the usual six-fold rotational symmetry was reduced to twofold. Interestingly, discreteness of grid cell firing returned if the animal roamed over a wall not by standing on pegs, but rather clinging to chicken wire so that the body was now aligned to the surface the animal was traversing [52]. However, the scale of the fields was greatly increased and the pattern became so large that its usual hexagonal symmetry could no longer be discerned. The speed-sensitive neurons in that region showed a blunted sensitivity to speed, suggesting that the expansion of the pattern might be because the cells were getting reduced information about distance travelled in a given time frame.

The symmetry of grid cell firing also breaks down when animals move in a volumetric space, as has been shown in two different species: bats [53] and rats [54]. Both experiments revealed similar findings in as much as the grid cells produced discrete fields but these were irregularly distributed throughout the volume; this irregularity appeared completely random for the rats but retained a local order in the bats, in which inter-field distances were more similar than expected by chance. Other than this small difference, the general similarity of the findings is quite striking because the movement patterns of these animals were differently constrained by the environment, in that the bats could move uninterruptedly in any direction whereas the rats were restricted by the bars of the lattice to move along orthogonal corridors. The difference between species—local order in the patterns in bats but not rats—might be explained not by fundamental evolutionarily conferred physiological differences, but rather by the different self-organization dynamics operating on fundamentally similar systems. Other physical systems can also show varying degrees of local order arising from the same basic substrate subjected to slightly different conditions (e.g. silicon dioxide which can be quartz, glass or amorphous: [55]).

## 3. Discussion: implications of firing symmetries for spatial coding

We have seen that the canonical spatial neurons show varying degrees of rotational and/or translational symmetry in their spatial firing patterns, arising from an interaction between intrinsic dynamics and external environmental influences. The question arises as to whether these properties have implications for spatial coding. *A priori*, spatial symmetries in neural firing could potentially have both negative and positive consequences on spatial coding.

The asymmetry of place cell firing in a single symmetrical environmental compartment is useful because it carries spatial information that disambiguates animal's current location, and indeed a spatial information measure has long been used to quantify this disambiguation [56]. In repeating environments when the place cell pattern repeats, spatial information is thus lower, and a decoding analysis (where the firing pattern is used to infer the animal's current location, based on previous mapping of place fields) yields multiple locations [13]. The repetition suggests that place cells are not using an obvious possible source of disambiguation, the distance travelled between one compartment and the next—this may be because of the observation by Carpenter *et al*. mentioned earlier that the grid cells themselves, which may convey distance information to place cells, repeat their patterns, at least until the environment is familiar [42]. This ambiguity in coding is associated with reduced spatial performance because animals have difficulty discriminating the compartments when they are aligned [14]. However, this difficulty disappears when the compartments are rotated so that they lie along different directions, which as we saw earlier also causes place cells to map the compartments differently. In general, then, it seems that place cells, and by implication spatial mapping by the animals as a whole, function best in conditions where asymmetry is maximized. However, the ambiguity arising from symmetries in firing may also serve a function, enabling generalization between similar spaces, allowing for more efficient coding and for learning occurring in one region of space to be applied in other, similar-looking regions.

With grid cells, the compelling symmetry of the grid pattern, which is intrinsic, immediately suggests that symmetry is the *point* of grid cells, and much work has gone in to trying to understand what that point may be. The translational symmetry of the grid means that the information carried by the firing of a single cell is spatially ambiguous, as with the repeating place fields described above. Any system looking only at that cell would have uncertainty about spatial location unless it also knew about the firing field inhomogeneities mentioned earlier [47]. However the superposition of grids at different scales removes this ambiguity for most practical purposes since the scale over which the entire ensemble pattern would repeat is many metres [38].

This symmetry-breaking in grid cells then raises the question of why there is symmetry in the first place. One possibility is that the cyclicity of the repeating pattern allows for conservation of neural resources. For example, if distance were to be tracked by firing rate then as distance increased, firing rates would steadily increase and so would energy usage, and the process would eventually reach a limit. By oscillating rates up and down instead, and using this information to drive some kind of counter, the unbounded linear quantity of distance can be turned into a bounded rotary signal instead. Of course, the counter itself steadily accumulates signal, albeit at a slower rate, but it may be that this is also cyclic (and may even comprise other grid cells).

It may be alternatively that the symmetry of grid cells actually has no function: that it is a by-product of whatever process causes the cells to fire in spatially discrete regions of uniform size. The characteristic hexagonal pattern appears when animals forage uniformly over the surface of a symmetrical, open arena, but we have seen that the pattern readily distorts if the arena is not symmetric [48] or if the trajectory of the animal is interrupted [35,51]. When we consider that the natural world is mostly asymmetric and full of barriers, it seems likely that a normal ‘day in the life of a grid cell’ does not entail producing a hexagonal close-packed array of firing fields. This consideration suggests, instead, that the relevant feature of grid firing is not in fact the symmetry. This is supported by the observations in 3D volumetric spaces described earlier that even when animals can explore the space using uninterrupted trajectories (as in bats) the fields are irregularly dispersed [53]. Since this same pattern was seen in rats, in an apparatus for which efficient navigation has been demonstrated [57,58], the implication is that regular grids are not needed for the spatial computations underlying navigation. This raises the question of what the relevant feature of grid cell firing is in that case: it seems likely that it must be the other salient characteristic, which is the discreteness of the firing regions. It may be that the primary function of grid cells is to discretize the space, perhaps to allow events in nearby regions of the space to be independently maintained in memory without mutual interference.

With head direction cells, as with place cells, it is initially the rotational asymmetry that is salient, because it imposes a polarization on an otherwise rotationally symmetric environment: this then lays the foundation for the other asymmetric signals such as the place and grid cells. The translational symmetry seen within a single compartment, called parallax correction [59] because the firing directions are the same everywhere in the space despite the apparent change in landmark direction (parallax) arising from the change in viewpoint, enables spatial positions to be computed in a stable position-independent orientational reference frame. The continuation of this parallel alignment across connected environments [16,17] presumably enables a global representation of space that enables computation of navigational trajectories across compartments (although this has yet to be demonstrated experimentally).

However, the sub-class of ‘multi-directional’ neurons described earlier, found in dysgranular retrosplenial cortex, produce two forms of rotationally symmetric firing patterns that may have a function. For between-compartment symmetry the pattern is only symmetric when the entire multi-compartment space is considered: the firing in each individual, asymmetric (polarized) compartment is asymmetric in the usual way, except that the firing direction follows the compartment layout rather than being fixed to the global reference frame. For the within-compartment symmetry the symmetric pattern is evident in each sub-compartment as well. A possible explanation is that the former group of cells are sensitive to environment layout and insensitive to global direction, while the latter receive inputs from both environment cues and the global direction cells—i.e. the head direction cells [30]. Since there are two (or four) sets of relationships between the local and global cues, associations form with two (or four) sets of head direction cells, and the cell will thus fire in two (or four) directions. Firing in one of the directions is usually a little stronger, and the assumption is that this was the direction corresponding to the conjunction of environment and global cues that was first experienced, and perhaps pre-existing, whereas the associations with the other directions were learned during exploration.

**Figure 6.**
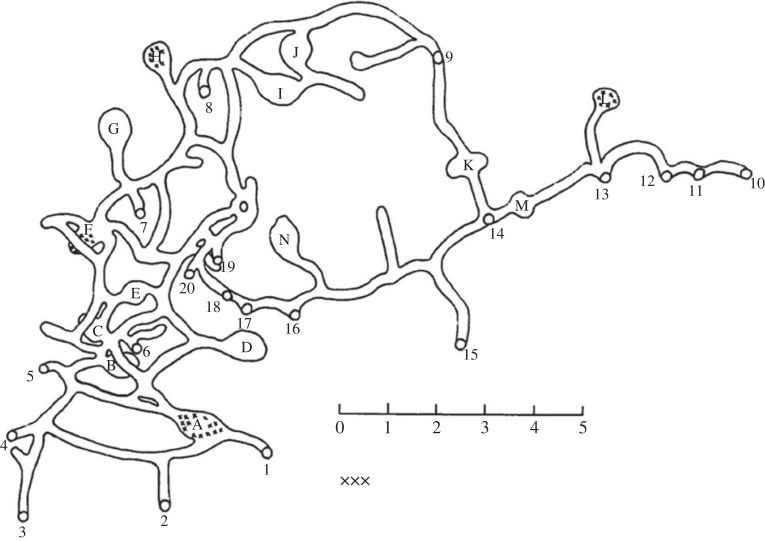
Burrow system of a colony of Norway rats studied by Calhoun [60] (adapted from figure on p. 29), showing repeating sub-compartments.

This rotational firing symmetry of the multi-directional neurons and egocentric boundary neurons discussed earlier might represent a confused state in which the neurons have incomplete information about facing direction; much like the translational symmetry confusion discussed earlier for place cells. However, also as suggested for place cells, such symmetries in firing may be beneficial, allowing for coding efficiencies in which the same firing pattern is recruited for the same set of environmental features even though these are occurring in different places, and/or at different orientations (a type of generalization). In particular, the fact that within-compartment multi-directional cells capture global environment symmetry and express it locally means that the system is instantaneously signalling information about the space beyond what the animal can see at that moment. One could think of the individual tuning curves of multidirectional neurons and egocentric boundary neurons as motifs, which repeat according to the environment layout and perhaps serve to signal to some other system what the global environment layout is. For animals that live in environments with repeating substructures—and rats fall into this category due to their complex 3D burrow systems [60] (figure 6)—this could be a useful way to encapsulate the global layout. If different cells are sensitive to different aspects of the visual scene, and each imprints (via its association with head direction cells) the orientations at which these aspects recur, the firing of the cell population would capture, locally, the layout of the global complex environment: something that may be useful for navigational planning.

The above argument about coding efficiency suggests that mirror symmetry would also be useful for a spatial system to encode. So far, there have been no reports of mirror-symmetric firing patterns in spatial neurons. However the visual system is sensitive to symmetry [61] and this is colour-specific [62], which suggests the operation of some kind of visual snapshot. It might therefore be that scene-processing regions such as the parahippocampal region (primates [63]) or postrhinal cortex (rodents [64]) will turn out to have neurons responsive to spatial mirror symmetry, which would support the notion that symmetry encoding is useful for optimizing spatial mapping.

A final point is that the issue of spatial symmetry in neuronal firing raises the larger issue of the role symmetry plays in spatial coding in humans. Many buildings have symmetry, in any of the domains (translational, rotational, mirror). The effect of environment symmetry on human wayfinding has not been extensively investigated, but the theoretical considerations outlined here suggest that it could potentially be helpful in facilitating spatial inference.

## Data Availability

This article has no additional data.
